# Effectiveness of Breathing Exercises and Stabilization Techniques on Patients with COVID-19: Quasi-Experimental Study

**DOI:** 10.3390/jcm14217730

**Published:** 2025-10-30

**Authors:** Jae Hyu Jung, Jung Wook Lee, Jin Young Ko

**Affiliations:** 1 Department of Occupational Therapy, Gyeonggi Provincial Medical Center, Anseong 17568, Republic of Korea; jj1258@naver.com; 2 Department of Physical Therapy, Gyeonggi Provincial Medical Center, Anseong 17568, Republic of Korea; 712leejw@naver.com; 3 Department of Rehabilitation Medicine, Seoul National University Bundang Hospital, Seongnam 13620, Republic of Korea

**Keywords:** physical therapy, respiratory exercises and stabilization techniques, pulmonary rehabilitation, mental health

## Abstract

**Objectives**: To evaluate the effects of respiratory exercises and stabilization techniques on patients with coronavirus disease 2019 (COVID-19). **Methods**: This is a quasi-experimental study and a single community-based hospital. Thirty-nine patients with COVID-19 completed the study, of which 20 underwent respiratory exercises and stabilization techniques, while the rest (19 patients) did not. Respiratory exercises and stabilization techniques comprised diaphragmatic breathing, pursed-lip breathing, square box breathing, huff coughing, and progressive muscle relaxation. Outcomes were assessed using the Modified Borg Scale (MBS), the Breathlessness, Cough, and Sputum Scale (BCSS), and the Hospital Anxiety and Depression Scale (HADS) for mental health. Questionnaires were administered at admission, discharge, and 6 months after discharge. **Results**: Patients who received respiratory exercises and stabilization techniques showed significant improvements in MBS (*p* = 0.0001) and BCSS scores (*p* = 0.002) at discharge. However, there were no significant long-term effects on these physical symptoms. Significant interaction in HADS scores were also observed. **Conclusions**: Although there were no significant long-term effects, respiratory exercises and stabilization techniques resulted in short-term improvements in the physical and mental health of patients with COVID-19.

## 1. Introduction

Coronavirus disease 2019 (COVID-19) is a respiratory disease, with 80% of patients experiencing upper respiratory tract infections [1]. A study observing patients 2 months after diagnosis reported persistent dyspnea in 53%, ongoing cough in 34%, and symptomatic improvement on chest radiography in only 27% [2]. In addition to respiratory complications, patients with COVID-19 often have psychological problems, including anxiety [3,4]. Dyspnea itself may contribute to anxiety by increasing energy expenditure [3,4].

Breathing exercises and stabilization techniques are widely used in pulmonary disease and can increase overall exercise performance by restoring normal diaphragmatic function, reducing dyspnea and respiratory rate, maintaining airway stability [5,6], and alleviating psychological problems [3,4].

Breathing exercises are widely used as non-pharmacological interventions that help improve thoracic expansion, strengthen respiratory muscles, and enhance oxygen saturation, thereby aiding in the recovery of respiratory function [7]. In addition, stabilization techniques promote core stability, which facilitates more efficient function of the respiratory muscles and contributes to improved overall physical coordination [8]. Patients with weakened respiratory muscles experience fatigue and shortness of breath, which interfere with daily functioning and require significant effort to overcome [9]. Furthermore, the World Health Organization (WHO) recommends educating COVID-19 patients about breathlessness and monitoring exercise intensity [10]. Therefore, specific techniques such as diaphragmatic breathing, intercostal breathing, and pursed-lip breathing, as well as trunk stabilization exercises, are expected to have positive effects not only on the respiratory recovery of COVID-19 patients but also on their rapid return to daily life.

Although previous studies have applied respiratory rehabilitation to patients with COVID-19, most of them have focused on outpatients [4] or home-based respiratory muscle training. In other words, they primarily targeted post-COVID-19 patients, and studies involving acute-phase patients remain limited [11,12].

Therefore, in this study, we aimed to confirm the effectiveness of respiratory exercises and stabilization techniques by applying them to hospitalized patients diagnosed with COVID-19. Breathing exercises and stabilization techniques are expected to effectively improve breathing and reduce anxiety and depression in patients with COVID-19 with low oxygen saturation. This study aimed to examine the effects of breathing exercises and stabilization techniques on respiratory symptoms and anxiety in patients with COVID-19.

## 2. Materials and Methods

### 2.1. Study Design and Setting

This quasi-experimental study was conducted in a single community-based hospital. The study was approved by the Institutional Review Board of Seoul National University Bundang Hospital and was classified as having minimal risk (IRB number: B-2110-713-303). Written informed consent was waived; instead, participants reviewed the study description and informed consent information via message and indicated their agreement by clicking the “I agree” button in the provided Google Form. The trial was registered with the Korea Clinical Trials Registry (KCT0006649). Eligible patients were sequentially allocated to the experimental and control groups in a 2:1 ratio based on the order of admission. Although a 2:1 ratio is sometimes used in randomized trials, in this study the allocation was predictable and therefore not truly random. The study was initially registered as a randomized controlled trial; however, because randomization was not feasible in practice, the design is more accurately described as quasi-experimental. This revision was made to reflect the actual study implementation and to ensure methodological accuracy.

### 2.2. Participants

We recruited patients with confirmed COVID-19 between 19 September 2021, and 4 May 2022. Inclusion criteria were as follows: (1) diagnosed with COVID-19 and isolated in the facility; (2) oxygen saturation below 95% or receiving oxygen therapy; (3) no history of psychiatric disorders; (4) no communication problems; (5) ≥18 years old; and (6) understood the research purpose and voluntarily agreed to participate. Exclusion criteria were as follows: (1) not medically stable, defined as requiring transfer to an intensive care unit, experiencing hemodynamic instability, or a rapid deterioration in respiratory status (e.g., oxygen saturation <90% despite oxygen therapy); (2) difficulty communicating in Korean; and (3) enrollment in another study.

### 2.3. Sample Size and Allocation

An a priori analysis was conducted using G*Power 3.1.9.7 software with a medium effect size (f = 0.25), α = 0.05, and power = 0.95. A total of 64 participants were required to detect significant between-group differences. A dropout rate of 20% was assumed; therefore, the study was oversampled accordingly.

Patients were assigned to the experimental or control group in a 2:1 ratio based on the order of admission.

### 2.4. Interventions

In this study, the intervention was categorized into breathing exercises and a stabilization technique. Breathing exercises included diaphragmatic breathing, pursed-lip breathing, square-box breathing, and huff coughing, which primarily target respiratory mechanics, airway clearance, and ventilation efficiency. The stabilization technique referred to progressive muscle relaxation (PMR), which was implemented to promote stress reduction, alleviate tension, and enhance psychological stability during the acute phase of illness. The program required no special equipment other than a patient handout and therapist instructions and was delivered in isolation wards by a licensed physical therapist with more than five years of experience in respiratory and rehabilitation therapy. It consisted of four breathing exercises—diaphragmatic breathing, pursed-lip breathing, square-box breathing, and huff coughing—and one stabilization technique, progressive muscle relaxation, with detailed procedures described in the subsequent text. The intervention was provided in person, with a therapist supervising the morning 30-min session and patients practicing independently in the afternoon 30-min session, for a total of 60 min per day over a two-week period. The exercises were standardized but could be adjusted in intensity based on patient tolerance, and adherence was monitored through therapist observation and patient self-report.

The first method was diaphragmatic breathing. In the lying position, one hand was placed on the chest and the other hand on the stomach. Deep breaths were taken through the nose to inflate the stomach. The lips were pursed while the participant exhaled slowly. The chest was kept as still as possible, and only the abdomen was allowed to rise. The second method was pursed-lip breathing. Participants relaxed the neck and shoulder muscles while inhaling slowly through the nose for two counts. The lips were pursed (as if whistling) while the participant exhaled slowly for four counts. The third method was square-box breathing. With eyes closed, participants inhaled through the nose for a count of four, held their breath for four counts, exhaled slowly for four counts, and paused for another four counts before repeating the cycle. The fourth method was huff coughing. In a seated position, participants took a slightly deeper breath than usual through the mouth, activated their abdominal muscles, and exhaled in three short bursts while making “ha,” “ha,” and “ha” sounds. The last method was progressive muscle relaxation. Participants assumed a comfortable position, took a few deep breaths, and tensed specific muscle groups before exhaling and releasing the tension. This was sequentially performed for the upper extremities, shoulders, head, neck, chest, abdomen, and lower extremities.

Both groups received standard conservative treatment according to COVID-19 response guidelines, with medical staff providing care based on each patient’s condition. A physical therapist administered the intervention, and an occupational therapist collected the data.

### 2.5. Outcome Measurements

Assessments were performed at admission, discharge, and 6 months after discharge.

The Modified Borg Scale (MBS) was used to measure the level of perceived breathlessness and dyspnea, with scores ranging from 0 (no breathlessness) to 10 (maximum breathlessness) [13].

The Breathlessness, Cough, and Sputum Scale (BCSS) is a three-item questionnaire used to assess dyspnea, cough, and sputum production on a 5-point Likert scale ranging from 0 (no symptoms) to 4 (severe symptoms) [14].

The Hospital Anxiety and Depression Scale (HADS) is a self-report questionnaire used to evaluate psychological problems in patients with medical illnesses. It comprises 14 items, with seven items each evaluating anxiety (HADS-A) and depression (HADS-D), each scored on a 4-point Likert scale (0–21 per domain) [15]. Scores are categorized as normal (≤7), borderline (8–10), and clinically significant (≥11) [16]. The Cronbach’s alpha values were 0.89 for anxiety and 0.86 for depression in Korea [17].

Hand grip strength was measured using a hand dynamometer (Jamar Preston, Jackson, MI, USA). It was assessed in both hands with the elbow at 90° flexion and the forearm and wrist in a neutral position. Three attempts were performed, and the highest value was recorded [18].

### 2.6. Statistical Analysis

Descriptive analysis was used to summarize continuous variables as means and standard deviations, and categorical variables as frequencies. The independent *t*-test for continuous variables and the chi-square or Fisher’s exact test for categorical variables were used to compare the characteristics of the two groups at baseline. Normality of continuous variables was tested using the Shapiro–Wilk test.

A linear mixed model (LMM) was used to compare the changes over time in each variable (MBS, BCSS, and HADS) between groups. The intervention effect across admission, discharge, and 6 months after discharge was analyzed using LMM. Statistical significance was set at *p* < 0.05. All analyses were conducted using SAS 9.4 statistical software (SAS Institute Inc., Cary, NC, USA).

## 3. Results

### 3.1. Participant Flow

**Figure 1:**
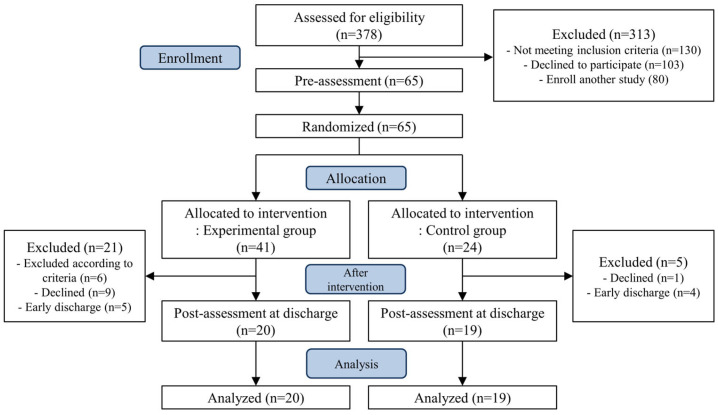
CONSORT flow diagram.

Of the 41 patients assigned to the experimental group, six were excluded based on the exclusion criteria during the intervention that move to intensive care unit or transfer to university hospital due to deteriorating health, nine chose to discontinue, and five were discharged earlier than expected and were unable to complete all sessions. Of the 24 patients in the control group, one withdrew from the study and four were discharged earlier than expected. Finally, 39 patients completed the study, of whom 20 completed respiratory exercises and stabilization techniques (Figure 1).

### 3.2. Patient Demographics

The experimental group consisted of 12 males and 8 females, with a mean age of 61.4 ± 12.26 years and a mean isolation duration of 13.25 ± 3.02 days. The control group included 9 males and 10 females, with a mean age of 67.37 ± 9.62 years and a mean isolation duration of 12.84 ± 3.66 days. There were no statistically significant differences between the two groups of patients regarding sex, age, duration of isolation, underlying diseases, or clinical symptoms (*p* > 0.05) (Table 1).

### 3.3. Outcome Measures

**Figure 2:**
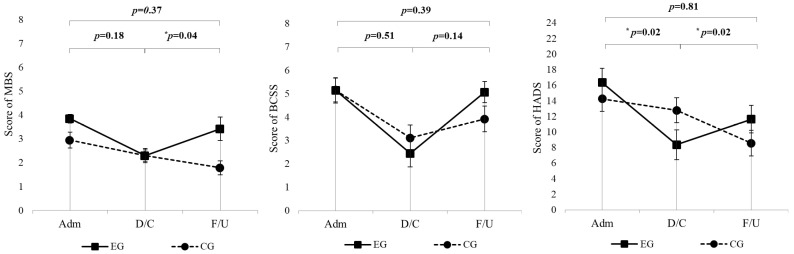
LMM used for longitudinal analysis of MBS, BCSS, and HADS scores over 6 months in the EG (*n* = 20) and CG (*n* = 19). All *p* values represent the time × group interaction. For each outcome, the top *p* value refers to the overall change from admission to 6-month follow-up, whereas the lower *p* values correspond to admission–discharge and discharge–6-month changes, respectively. EG = experimental group; CG = control group; MBS = Modified Borg Scale; BCSS = Breathless, cough, and sputum scale; HADS = Hospital Anxiety and Depression Scale; Adm = Admission; D/C = Discharge; F/U = 6-month follow-up. * *p* < 0.05.

The MBS scores did not show significant interaction effects between time points and groups (*p* = 0.18) (Table 2, Figure 2). In the experimental group, MBS scores significantly improved at discharge compared to admission (*p* = 0.0001) (Table 3). Additionally, there were significant differences in MBS scores between the experimental and control groups at admission (*p* = 0.02), but this difference disappeared at discharge (*p* = 0.48) (Table 4). BCSS scores across the study period did not demonstrate significant interaction effects between groups and time points (*p* = 0.51) (Table 2, Figure 2). The experimental group exhibited significant improvements at discharge (*p* = 0.002), with scores also significantly better than those of the control group at this time point (*p* = 0.002) (Table 3). For the HADS scores, significant interaction effects were observed (*p* = 0.02), with the experimental group showing greater reductions in scores from admission to discharge compared to the control group (Table 2, Figure 2). Significant reductions were also noted in the experimental group at both discharge (*p* = 0.0002), and 6-month follow-up (*p* = 0.03). However, no significant differences were observed in the control group (Table 3). Hand grip strength did not differ significantly between groups at admission or discharge (all *p* > 0.05), and no clinically meaningful between-group changes were detected over the hospitalization period (Table 1).

## 4. Discussion

Our study found that respiratory exercises and stabilization techniques significantly improved MBS and BCSS scores at discharge, demonstrating its effectiveness in reducing breathlessness and respiratory symptoms in patients with COVID-19. While there were no significant long-term effects on these physical symptoms, significant reductions in HADS scores were observed, indicating substantial short-term improvements in mental health. However, these benefits did not persist at the six-month follow-up, highlighting the need for continued intervention.

According to previous studies, patients with COVID-19 often continue to experience respiratory symptoms even after discharge [19], which may affect their exercise capacity and mental health [3,4]. Therefore, breathing exercises and stabilization techniques have been applied to reduce dyspnea and improve the mental health of these patients. Similarly to Liu et al. [3,4], our study observed significant improvements in respiratory function and psychological well-being shortly after the intervention. However, unlike Liu et al., who reported prolonged mental health benefits, our study did not observe these long-term effects. This discrepancy could be attributed to the differences in the intensity and duration of the respiratory exercises and stabilization techniques programs. Liu et al. implemented a comprehensive rehabilitation regimen that extended beyond the immediate post-recovery phase, potentially contributing to the lasting benefits observed in their study. Respiratory muscle training and stretching exercises were performed in addition to the breathing exercises in our study, and the intervention period was much longer at 6 weeks. In a study by Jimeno-Almazán, dyspnea and fatigue were significantly improved in the group that received both the exercise program and inspiratory muscle training compared to the group that received only inspiratory muscle training [20]. Therefore, the composition and duration of the intervention may have influenced the study results, and the importance of exercise and breathing exercises in respiratory exercises and stabilization techniques should be considered.

Patients with COVID-19 complain of anxiety and depression during and after isolation [21,22]. Respiratory rehabilitation had a significant effect on anxiety and depression in this study. These results are similar to those reported by Liu et al. [3,4]. who reported a significant improvement in relaxation treatment, suggesting an improvement in mental health, which is consistent with the findings of Jimeno-Almazán et al. However, these benefits did not persist long-term, emphasizing the need for interventions even after discharge to maintain mental health.

Our findings have several implications for clinical practice. First, while short-term interventions are beneficial, they may not be sufficient to produce enduring benefits, particularly in mental health. It is crucial for rehabilitation programs to include follow-up care, potentially with remote or home-based components, to support patients post-discharge.

Second, integrating multidisciplinary approaches, such as including physical therapists, occupational therapists, and psychologists, can enhance the holistic benefits of respiratory exercises and stabilization techniques. Such approaches have been shown to improve both physical and psychological outcomes in patients with chronic conditions and may be particularly effective in the context of COVID-19, where patients often experience multifaceted health challenges.

This study has some limitations. First, the intervention period during hospitalization was too short, and no continuous intervention was provided after discharge. This may have affected the ability to capture long-term outcomes, especially given that outcomes were followed for 6 months while the intervention lasted only 2 weeks. Future research should consider longitudinal studies with extended follow-up durations to better understand the long-term impacts of respiratory exercises and stabilization techniques. Second, an objective evaluation of respiratory function could not be conducted, which may have introduced bias as the evaluation relied on the patient-reported outcomes via subjective questionnaires. Third, although 64 patients were initially enrolled and assigned to the experimental or control group in a 2:1 ratio, a larger number of patients in the experimental group were excluded due to deterioration in clinical condition (e.g., transfer to an intensive care unit or university hospital), voluntary discontinuation, or early discharge before completing the intervention. As a result, 39 patients (20 in the experimental group and 19 in the control group) were included in the final analysis. Such attrition is common in studies involving acute-phase patients and may have reduced the statistical power and generalizability of the findings, increasing the risk of Type II error. Importantly, the dropout rate was higher in the experimental group than in the control group. This pattern is not unexpected, as patients required to perform additional structured exercises are often more likely to discontinue participation, particularly in acute-care settings where clinical status can change rapidly. Nevertheless, the final cohorts were comparable in baseline characteristics and group size, and the comparison between 20 and 19 patients still provides meaningful evidence regarding the intervention effect.

## 5. Conclusions

Respiratory exercises and stabilization techniques may provide short-term benefits for respiratory symptoms and psychological well-being in patients with COVID-19. However, due to the small sample size, potential biases, and the impossibility of blinding in this study design, the findings should be interpreted with caution. Future studies with larger cohorts, rigorous designs, and extended follow-up are needed to confirm the effectiveness and long-term impact of these interventions. 

## Figures and Tables

**Table 1:** Demographic and clinical characteristics according to groups.

Classification		EG (*N* = 20)	CG (*N* = 19)	*p*
Sex, *n* (%)	Male	12 (60%)	9 (47.37%)	0.43
	Female	8 (40%)	10 (52.63%)	
Age (year), M (SD)		61.4 (12.26)	67.37 (9.62)	0.31
Duration of isolation (day), M (SD)		13.25 (3.02)	12.84 (3.66)	0.42
Rt. HP at admission		22.37 (2.75)	21.77 (1.88)	0.09
Lt. HP at admission		21.03 (2.52)	21.31 (2.00)	0.28
Rt. HP at discharge		22.90 (2.39)	21.91 (1.79)	0.17
Lt. HP at discharge		23.46 (2.50)	22.81 (1.81)	0.12
Underlying disease, *n* (%)	HTN	8 (40%)	10 (52.63%)	0.43
	DM	10 (50%)	5 (26.32%)	0.13
	Dyslipidemia	6 (30%)	5 (26.32%)	0.79
	CVD	1 (5%)	0 (0%)	+0.51
	CAD	3 (15%)	3 (15.79%)	+0.34
	Etc.	1 (5%)	3 (15.79%)	+0.24
Vaccinated before infection		16 (80%)	13 (68.42%)	0.21
Clinical symptoms, *n* (%)	Fever (over 37.5 °C)	13 (65%)	12 (63.16%)	0.90
	Pneumonia	18 (90%)	17 (89.47%)	+0.40
	Myalgia	9 (45%)	5 (26.32%)	0.22
	Chilling	8 (40%)	6 (31.58%)	0.58
	Cough & sputum	18 (90%)	16 (84.21%)	+0.32
	Fatigue	0 (0%)	1 (5.26%)	+0.49
	Headache	9 (45%)	4 (21.05%)	0.11
	Sore throat	9 (45%)	4 (21.05%)	0.11
	Nausea & vomiting	6 (30%)	3 (15.79%)	+0.18
	Diarrhea	4 (20%)	0 (0%)	+0.06
	Dyspnea	15 (75%)	14 (73.68%)	+0.28
	Hyposmia & hypogeusia	1 (5%)	1 (5.26%)	+0.51
	Dizziness	1 (5%)	1 (5.26%)	+0.51

EG = experimental group; CG = control group; M = mean; Rt. = right; Lt. = left; HP = hand power; HTN = Hypertension; DM = diabetes; CVD = cerebrovascular disease; CAD = coronary artery disease. +: Fisher’s exact test. Duration of isolation refers to the period from the quarantine start date to the discharge evaluation date.

**Table 2:** LMM results for outcomes during follow-up period (*N* = 39).

	MBS			BCSS			HADS (Total)			HADS (Anxiety)			HADS (Depression)		
	E	SE	*p*	E	SE	*p*	E	SE	*p*	E	SE	*p*	E	SE	*p*
Group															
EG	1.60	0.59	0.008 **	1.04	0.98	0.29	2.79	2.99	0.35	1.62	1.15	0.164	2.05	1.45	0.162
CG	Reference			Reference			Reference			Reference			Reference		
Time															
F/U	−1.16	0.56	0.04 *	−1.13	0.84	0.18	−5.81	2.11	0.009 **	−1.84	0.81	0.03 *	−3.74	1.16	0.003 **
D/C	−0.64	0.48	0.19	−2.03	0.74	0.01 *	−1.61	1.84	0.39	−0.26	0.81	0.75	−1.21	1.12	0.28
Adm	Reference			Reference			Reference			Reference			Reference		
Time × Group															
EG × F/U	0.70	0.76	0.37	1.05	1.15	0.39	0.71	2.89	0.81	0.49	1.13	0.67	1.09	1.63	0.51
EG × D/C	−0.90	0.67	0.18	−0.67	1.00	0.51	−6.39	2.55	0.02 *	−3.34	1.12	0.004 **	−3.19	1.56	0.045 *
CG × Adm	Reference			Reference			Reference			Reference			Reference		
EG × F/U	1.60	0.75	0.04 *	1.72	1.15	0.14	7.10	2.95	0.02 *	−3.83	1.13	0.001 **	−4.28	1.56	0.008 *
CG × D/C	Reference			Reference			Reference			Reference			Reference		

EG = Experimental group; CG = Control group; E = Estimate; SE = Standard error; MBS = Modified Borg Scale; BCSS = Breathless, cough, and sputum scale; HADS = Hospital Anxiety and Depression Scale; Adm = Admission; D/C = Discharge; F/U = 6-month follow-up. * *p* < 0.05, ** *p* < 0.001.

**Table 3:** Mental health outcome at admission, discharge, and outpatient follow-up within group.

			MBS			BCSS			HADS		
			Diff (A − B)	SE	*p*	Diff (A − B)	SE	*p*	Diff (A − B)	SE	*p*
EG	F/U	D/C	1.13	0.56	0.05	2.62	0.54	<0.001 **	4.71	1.68	0.01 *
		Ad	−0.42	0.51	0.42	−0.08	0.50	0.88	−3.30	1.44	0.03 *
	D/C	Ad	−1.55	0.33	<0.001 **	−2.70	0.73	0.002 *	−8.00	1.73	<0.001 **
CG	F/U	D/C	−0.60	0.39	0.14	0.81	0.34	0.34	−2.69	2.44	0.28
		Ad	−1.16	0.50	0.03 *	−1.24	0.79	0.13	−4.17	2.20	0.07
	D/C	Ad	−0.64	0.41	0.14	−2.05	0.58	0.002 *	−1.47	1.21	0.24

EG = Experimental group; CG = Control group; Diff = Difference; SE = Standard error; MBS = Modified Borg Scale; BCSS = Breathless, cough, and sputum scale; HADS = Hospital Anxiety and Depression Scale; Adm = Admission; D/C = Discharge; F/U = 6-month follow-up. * *p* < 0.05, ** *p* < 0.001.

**Table 4:** Mental health outcome at admission, discharge, and outpatient follow-up between group.

	Admission			Discharge			Outpatient f/u		
	EG M(SE)	CG M(SE)	*p*	EG M(SE)	CG M(SE)	*p*	EG M(SE)	CG M(SE)	*p*
MBS	3.85 (0.18)	2.95 (0.33)	0.02 *	2.30 (0.24)	2.31 (0.30)	0.48	3.43 (0.49)	1.79 (0.29)	0.02 *
BCSS	5.15 (0.54)	5.16 (0.51)	0.76	2.45 (0.58)	3.11 (0.55)	0.73	5.07 (0.45)	3.92 (0.55)	0.44
HADS	16.35 (1.84)	14.26 (1.62)	0.53	8.35 (1.90)	12.79 (1.58)	0.38	11.64 (1.76)	8.56 (1.64)	0.69

EG = Experimental group; CG = Control group; M = Mean; SE = Standard error; MBS = Modified Borg Scale; BCSS = Breathless, cough, and sputum scale; HADS = Hospital Anxiety and Depression Scale. * *p* < 0.05.

## Data Availability

The datasets used and/or analyzed during the current study are available from the corresponding author on reasonable request.

## Abbreviations

The following abbreviations are used in this manuscript:

BCSS	Breathlessness, Cough, and Sputum Scale
CG	Control group
COVID-19	Coronavirus Disease 2019
EG	Experimental group
HADS	Hospital Anxiety and Depression Scale
LMM	Linear Mixed Models
M	Mean
MBS	Modified Borg Scale
